# Two Handbooks on House Drainage

**Published:** 1892-05-07

**Authors:** 


### THE WORKSHOP BOOKSHELF.

### TWO HANDBOOKS ON HOUSE DRAINAGE.

1.—House Drainage. By G. A. T. Middleton. (London: P. T. Batsford, 52, High Holborn. 1892).

This little work professes to be a handbook for architects and building inspectors, and as far as it goes, fulfils its purpose simply and well. The author starts with laying down the main principles or essentials of good drainage, and then proceeds to discuss the various parts in detail. The main principles of good drainage are at the present day tolerably well known, though they are not by any means universally acted upon. That pipes should be laid on a solid foundation, that they should be made of impermeable material, well glazed inside and jointed together with water-tight joints, that the falls or gradients of the pipes should be regular and sufficient, that all waste pipes other than those used for the conveyance of fæcal matter should be aerially disconnected from the drains, and that free ventilation should be provided throughout the entire system, are rules which form the A B C of good drainage. These and other minor principles are clearly and simply stated, and are illustrated by two plans, one of a large country house, the other of a terrace house and shop, the drainage being in each case shown in detail.

Disconnection from the sewer pipes, and the methods of laying and jointing water closets, slop sinks, and urinals, rain water and waste pipes, and stable drainage are all treated in successive chapters, and the two last chapters deal respectively with drain testing and the way to write a specification.

The illustrations are nearly all good, and drawn in a practical, clear way, and though the author has evidently been unable to avoid illustrating the wares of particular makers, he has exercised on the whole a fair amount of discrimination and impartiality.

2.—The Drainage of Habitable Buildings. By W. Lee Beardmore. (London: Whittaker and Co. 1892.)

The author of this work holds some remarkable views as to the nature and scope of his subject. His first chapter, which he calls introductory, treats of the atmosphere [its composition and probable depth, of the construction of the human heart and the circulation of the blood, of respiration, of the nature of various kinds of gases, and of the germ theory of disease. And all this imposing array of science condensed into seven pages leads up to what? The necessity for a syphon trap between a house drain and a sewer! Truly, a case of *ridiculus mus*!

Towards the end of the book the author again "drops into" science, this time in connection with the disposal of sewage. Not content with pointing out the necessity of exercising due care in allowing cattle to feed off sewage-fed herbage, our author must needs favour us with a dissertation on tape-worm, measly beef, and the conditions under which the ova of parasites live and die.

These little eccentricities apart, there is much in the book that is sound and good, and much that would be more useful still if it were stated in a simpler form, and made clear by diagrams. The formulæ, for instance, for calculating the discharge of water through a drain is quite unsuitable for such a work as this. Far better would it have been to have given a short table showing the discharge in gallons per minute of four, six, and nine-inch pipes at, say, some dozen different gradients, than to give such a formula as that devised by Mr. Henry Robinson for calculating the discharge through sewers.

The illustrations are poor in quality and meagre in quantity; and the book would be greatly improved in practical value if the letterpress were condensed and the verbal embroidery cut out; and more and better diagrams introduced. Another great improvement we earnestly commend to the author is the careful elimination of the first person singular.

